# Preliminary Experience with a New Multidirectional Videoendoscope for Neuroendoscopic Surgical Procedures

**DOI:** 10.1371/journal.pone.0147524

**Published:** 2016-01-27

**Authors:** Luigi Maria Cavallo, Alberto Di Somma, Domenico Solari, Oreste de Divitiis, Umberto Marcello Bracale, Paolo Cappabianca

**Affiliations:** 1 Department of Neurosciences, Reproductive and Odontostomatological Sciences, Division of Neurosurgery, Università degli Studi di Napoli "Federico II", Naples, Italy; 2 Department of Public Health, Unit of Vascular and Endovascular Surgery, Università degli Studi di Napoli "Federico II", Naples, Italy; Emory University School of Medicine, UNITED STATES

## Abstract

**Purpose:**

We assessed the applicability of a new multidirectional videoendoscope (digiCAMeleon, Karl Storz GmbH, Tuttlingen, Germany) in various neuroendoscopic procedures.

**Methods:**

A 4-mm-diameter rigid videoendoscope (digiCAMeleon, Karl Storz GmbH, Tuttlingen, Germany) with 1 sensor and an internal LED light source was tested. The device offers a resolution of 1920 x 1080 pixels and weighs ≈ 215 g. The prototype was tested on three cadaveric heads using three different approaches: a) endoscopic endonasal transsphenoidal; b) frontal transcortical intraventricular; c) supraorbital.

**Results:**

We identified several major benefits of the integrated system as applied to endoscopic endonasal, transcortical intraventricular, and endoscopic supraorbital keyhole approaches. These included improved maneuverability of the scope on account of reduced bulk and integration of the camera and fiberoptic light components, a variable angle of view from 0–70 degrees, and a novel feature that can be activated to maintain orientation of the surgical horizon. Our preliminary report highlights the potential for handling the videoendoscope in one hand, as one would a microsurgical instrument. The videoendoscope harbors all its electronic and lighting data into a unique and thin cable, thus resembling a modern "all-in-one" computer technology. Because of its reduced weight and ergonomic shape, controlling its movements is very easy and comfortable, even in the microsurgical environment. Furthermore, the videoendoscope offers the unique feature of orienting the horizon of vision, thanks to the possibility of offering angled views while working; this helps the surgeons to stay oriented with direct visualization and improved control of the instruments over a specific area of interest.

**Conclusions:**

The videoendoscope prototype represents an HD-image quality versatile tool in a neurosurgical environment, thanks to its reduced weight and dimensions; in these preliminary simulations, we have identified optimized visibility and maneuverability as major benefits of this novel surgical adjunct.

## Introduction

The endoscope was first introduced in neurosurgery in the early decades of the 20th century and since then it has gradually become part of the neurosurgeon’s armamentarium, thanks to enormous technological progress and ongoing refinements. A new subspecialty in neurosurgery (i.e. neuroendoscopy) has developed as a result of the versatility and applicability of the neuroendoscope to a multitude of neurosurgical approaches. Neuroendoscopy, adopted in the early 1920's thanks to the contribution of pioneers, Walter Dandy [1] among all, has permitted neurosurgeons to access deep structures within both the cranial and spinal compartments, thanks to its panoramic views, proximity to the surgical target, and minimization of tissue retraction and brain manipulation [2].

On the other hand, the development of microneurosurgery in the 1960s initially limited the widespread use of the endoscopic technique, because of its then inferior quality of vision as compared to the microscope, which has consistently provided high magnification and adequate illumination while maintaining stereoscopic visualization. Although the 1960's represented a time during which publications on neuroendoscopy were sparse, it was also a time when key technological advances were made, laying the groundwork for the future reinassance of neuroendoscopy. These technological advances included the idea of variable refractive index lenses by Harold Hopkins (i.e. the SELFOC lens), the invention of charged-coupling devices (CCDs) and improved fiberoptic technology [3]. CCDs represented a new boost for endoscopy: this "machine" was designed to convert optical signals to electrical impulses, thus becoming invaluable tools for modern neuroendoscopes and advancing the miniaturization process. Finally, the introduction of advanced optical systems, which reduced loss of reflected light and tremendously improved video-capture methods, cultivated an increased interest in the field of neuroendoscopy over the last decades.

Despite the fact that the quality of endoscopic imaging reached an incredible high-quality standard only 20 years ago, one of the main limitations to its widespread use in neurosurgery still stems from drawbacks due to handling of the endoscope, cumbersomeness related to the camera and light cable connections, and maneuverability inside the skull. Different fiberscopes and rigid endoscopes have been proposed with smaller diameters and shorter lengths, including the latest videoendoscopes with the “chip stick” technology [4–6]. However, the downside of optimizing miniaturization and weight reduction has always been deterioration of the quality of vision. Unfortunately, in an era where HD-quality images are the standard and dominate both the endoscopic and microsurgical fields, inadequate image quality is no longer tolerable. According to these strict requirements, above all the need to couple miniaturization and high image quality, a new videoendoscope equipped with an HD chip was recently developed. The purpose of this study is to evaluate the main features, including both pros and cons, of this new rigid videoendoscope (digiCAMeleon, Karl Storz GmbH, Tuttlingen, Germany) as applied to a variety of neurosurgical procedures.

## Materials and Methods

### Anatomical Study Design

Anatomical dissections were performed at the Institute of Clinical Anatomy of the Eberhard-Karls-University, Tuübingen (Germany). This study was approved by the Ethics Committee of the Medical Faculty of the Eberhard-Karls-University of Tübingen (Germany). Written informed consent from the donor or next of kin was obtained for the use of all samples in research. Three pure endoscopic approaches were used in three human cadaver heads where the arterial system was pre-injected with red-colored latex: a) endoscopic endonasal transsphenoidal; b) frontal transcortical; c) supraorbital.

All dissections were run under surgical conditions with heads positioned to simulate the orientation used in the operating room. The new digiCAMeleon videoendoscope was the only visualization tool utilized. We evaluated the technical potential of the device in terms of visibility and maneuverability, aiming to identify the key neuro-anatomical structures from different standpoints.

### digiCAMeleon videoendoscope

**Fig 1:**
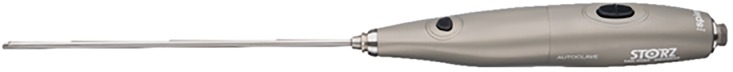
Image of the digiCAMeleon.

The 4-mm-diameter rigid videoendoscope (digiCAMeleon, Karl Storz GmbH, Tuttlingen, Germany) is an electronic version of the ENDOCAMELEON, with 1 sensor and an internal LED light source. This device offers a digital, continuously changing direction of view (DOV), i.e. between 0–70 degrees. It is equipped with an autorotation and offset mode, offers a resolution of 1920 x 1080 pixels, and weighs only 215 g (Fig 1, Table 1).

**Table 1:** Comparative analysis of the 3CCD-HD camera and the videoendoscope digiCAMeleon.

	HD CAMERA	VIDEOENDOSCOPE digiCAMeleon
*Image sensor*	IMAGE1 Three-Chip Camera Head 3 x 1/2 CCD chip	1 sensor and an internal LED light source
*Pixels*	1920 x 1080 (per chip)	1920 x 1080
*Weight*	246 g (camera) + 150 g (endoscope) + 50 g (cable) = 450 g (approx.)	215 g
*Frequency*	50–60 Hz	50–60 Hz
*Others*	2 programmable camera head buttons, cable (300 cm in leght) non-detachable, grip mechanism: standard eyepiece detector, SPIES compatible	Autoclavable, programmable camera head buttons, KARL STORZ IMAGE1 SPIES (Storz Professional Image Enhancement System) camera platform, 4-mm diameter rigid videoendoscope, autorotation and offset mode, digital, continuously change of DOV (direction of view) between 0–70 degree, electronic version of an EndoCAMeleon

The videoendoscope has an integrated image sensor that also includes an embedded light source, offering a free direction of viewing while the tip itself does not move. In this newly developed device, all the electronic and lighting data flow into a unique and thin cable, thus resembling a modern "all-in-one" computer technology.

The videoendoscope is connected to the KARL STORZ IMAGE1 SPIES (Storz Professional Image Enhancement System) camera platform, which features an innovative technology related to an improved image quality visualization system.

## Results

Using the videoendoscope it was possible to easily perform the endonasal approach to the sella, suprasellar and clival areas. Visualization of the ventricular cavities via a frontal transcortical route, as well as exposure of the optico-chiasmatic and optico-carotid regions via a supraorbital approach, were all possible and facilitated by the novel, integrated system.

One of the major benefits derived from our study is that the videoendoscope can be held like a microsurgical instrument in one hand. On account of its weight and ergonomic shape, iuse of the videoendoscope precisely facilitates control of movements in a microsurgical environment.

There are three buttons on the videoendoscope, one in front to switch direction of view (DOV) and two in the back to set the horizon of vision; the direction of view may change in a continuous manner from 0 to 70 degrees, or at preset variable intervals (0, 30, 45, 70 degrees). Furthermore, the new digiCAMeleon offers a new feature, the ability to set or changing the horizon of vision through the channels on the handle. This means that, by setting the horizon of vision, the orientation of the image is maintained automatically, even when turning the videoendoscope; on the contrary, rotation of the camera-endoscope system does affect the orientation of the image. This function helps maintain the correct orientation of vision regardless of rotation of the videoendoscope in the hand, and on the other hand it makes coaxial rotation of the surgical instrument around the video endoscope without changing the orientation of the image on the screen possible. This function is particularly advantageous during intraventricular procedures.

### Endoscopic endonasal approach

**Fig 2:**
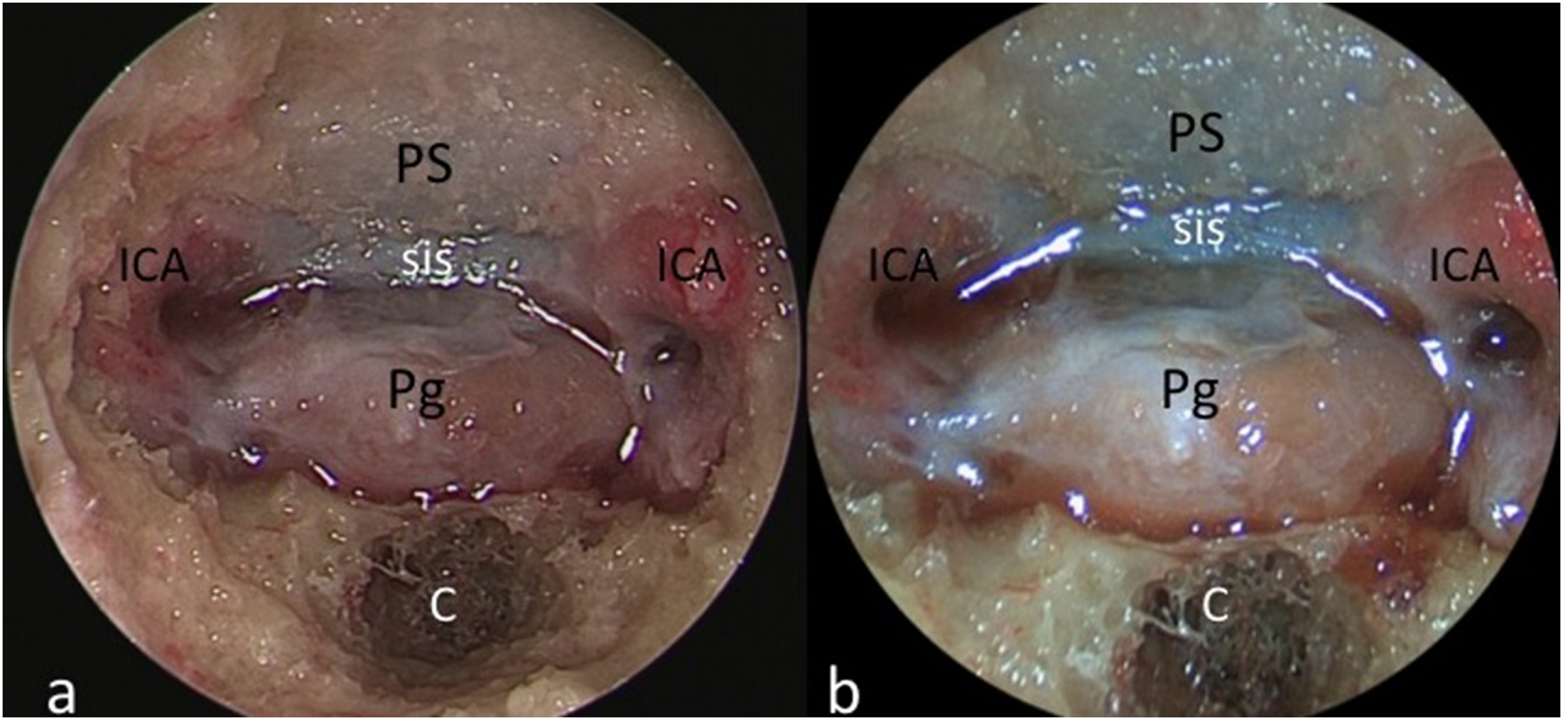
Comparative images of the endoscopic endonasal approach obtained with the HD-camera (a) and the videoendoscope (b). Exposure of the pituitary gland enclosed between the internal carotid arteries. The videoendoscope grants proper illumination in each part of field, in both high and low light (shadow) areas. *PS*: *planum sphenoidale; sis*: *superior intercavernous sinus; ICA*: *internal carotid artery; Pg*: *pituitary gland; C*: *clivus*.

**Fig 3:**
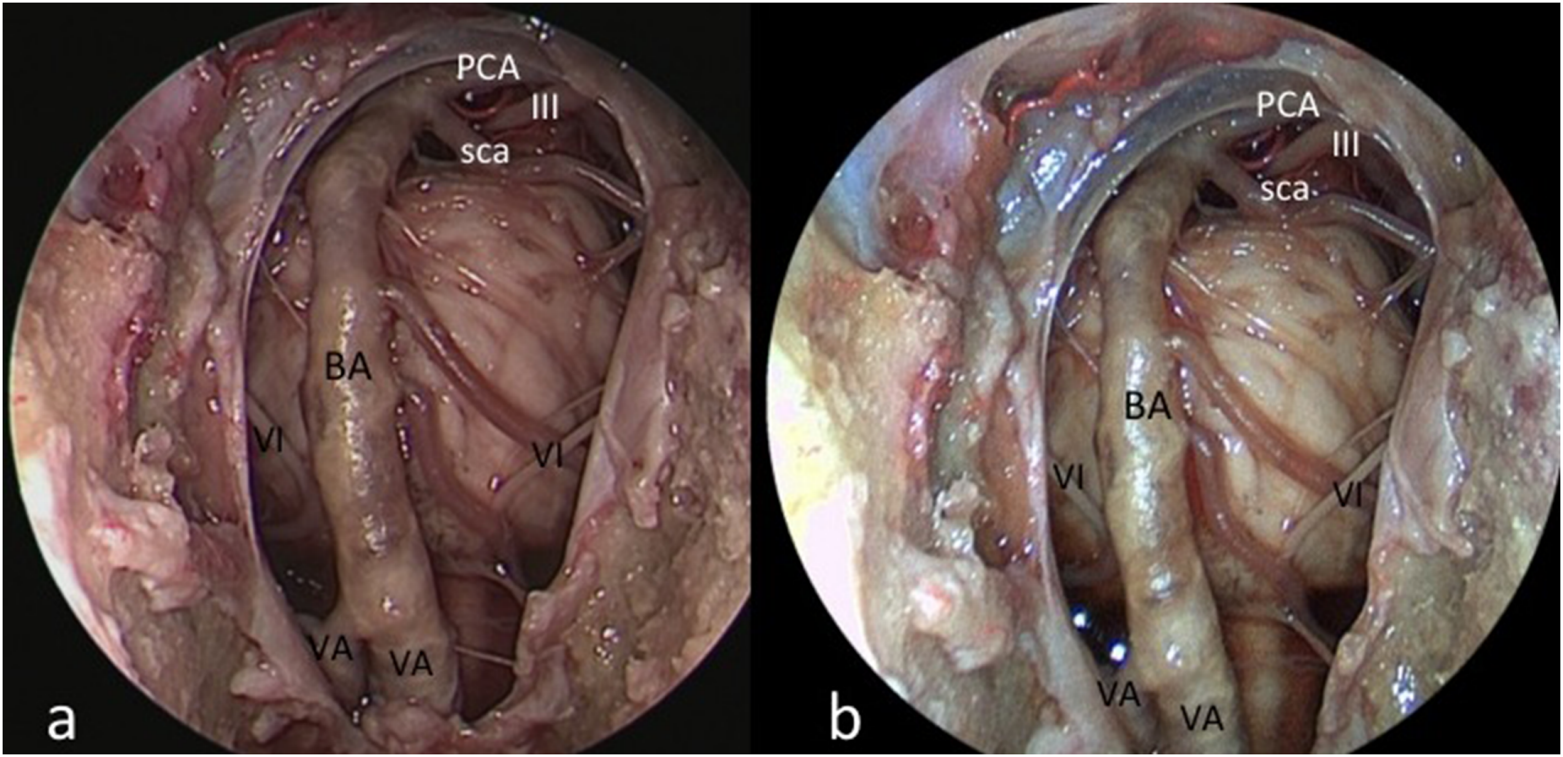
Endoscopic endonasal extended approach to the clivus; comparative images obtained with HD-camera (a) and the videoendoscope (b). BA: basilar artery; sca: superior cerebellar artery; III: oculomotor nerve; VA: vertebral artery; VI: abducent nerve.

In all the three specimens, it was possible to easily explore the nasal and paranasal structures (Fig 2). The procedure was extended to the suprasellar and clival regions, where the dura mater was opened to visualize the intradural neurovascular structures (Fig 3) [7, 8]. Use of the miniaturized and integrated videoendoscope offered less bulkiness while maintaining resolution and illumination quality. The auto-orientation feature was also useful in the endonasal approach in maintaining horizontal alignment of the sella and skull base.

### Transcortical intraventricular approach

**Fig 4:**
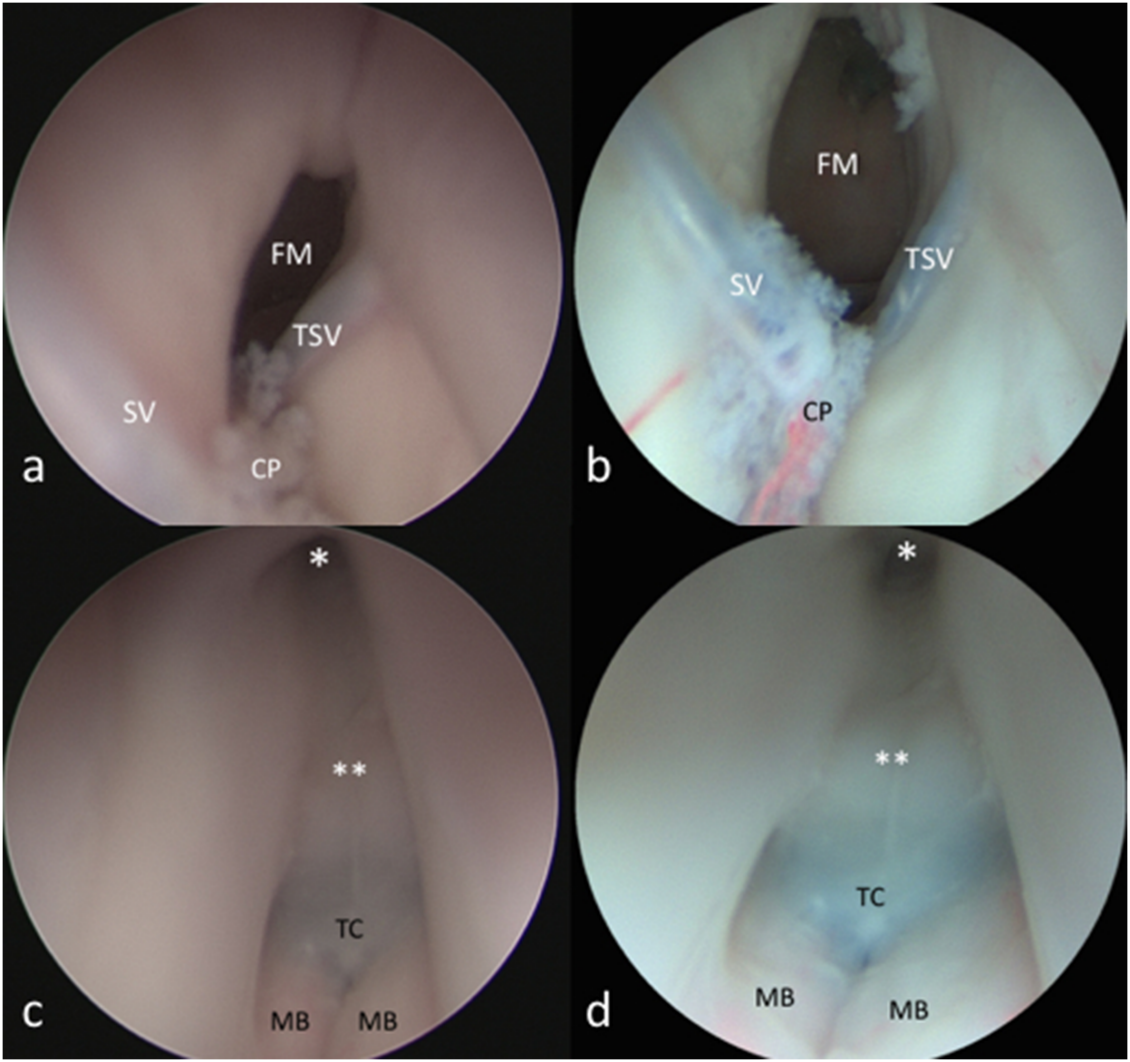
Frontal transcortical approach to the lateral and third ventricle. Picture obtained with the aid of the HD-camera (a, c) and the videoendoscope (b, d). *FM*: *foramen of Monro; CP*: *choroid plexus; SV*: *septal vein; TSV*: *thalamo-striate vein; MB*: *mammilary body; TC*: tuber cinereum; *: chiasmatic recess; **: *infundibulum*.

The great advantage we identified using the videoendoscope in this corridor was facilitate by its extreme maneuverability inside the ventricular cavity, almost resembling a rigid fiberscope but with much higher image quality. The ability to change the direction of view (DOV) from 0 to 70 degrees without moving the videoscope offers additional advantages in navigating the ventricular cavity and in the visualization of the floor of the third ventricle (Fig 4). During transventricular procedures, the possibility of setting the horizon of vision helps the surgeon to stay oriented while rotating the videoendoscope, in order to reach a specific area of interest with an instrument.

### Supraorbital approach

**Fig 5:**
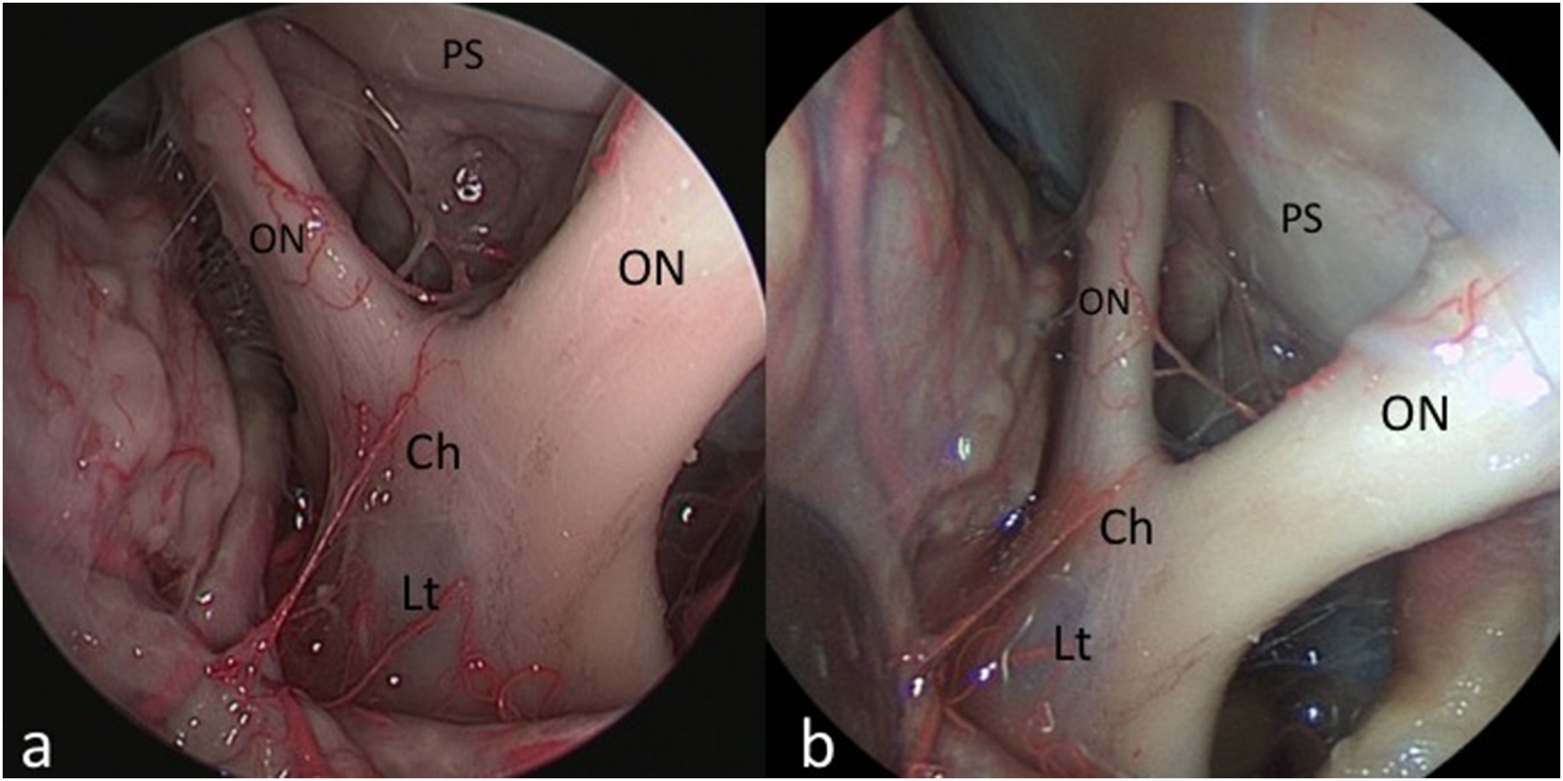
Supraorbital approach to the opto-chiasmatic region. HD-camera (a) and videoendoscopic views (b). *ON*: *optic nerve; Ch*: *chiasm; PS*: *planum sphenoidale; Lt*: *lamina terminalis; ICA*: *internal carotid artery*.”

The extreme versatility and maneuverability of the videoendoscope again facilitated intradural exploration via this corridor [9, 10]. The optico-chiasmatic region was clearly visualized and, due to minimization of its cumbersomeness and bulk, it was possible to introduce the scope through different surgical corridors, e.g., inter-optic, optico-carotid and carotid-oculomotor triangles (Fig 5).

## Discussion

Over the last several decades, technological progress led to tremendous improvement in terms of endoscopic image quality, but a similar boost in terms of miniaturization of camera-endoscope coupled devices was not realized. A few prototypes of rigid multidirectional endoscopes, based either on prism optics or video techniques, have been described in literature [11, 12].

The novel videoendoscope tested in this study was recently developed with the aim of optimizing miniaturization of the visualizing tool and maintaining high image quality. We identified several major benefits of the integrated system as applied to endoscopic endonasal, transcortical intraventricular, and endoscopic supraorbital keyhole approaches. These benefits included improved maneuverability of the scope on account of reduced bulk and integration of the camera and fiberoptic light components, a variable angle of view from 0–70 degrees, as well as a new feature that can be activated to maintain orientation of the surgical horizon. It represents the natural evolution of the recently proposed Endactive [11]. As compared to the rigid endoscope connected to a high-definition camera, the videoendoscope offers similar image quality with better handling and maneuverability inside the surgical field (see Table 1). In particular, this tool provides proper light enhancement in each part of the endoscopic field at a deeper target (see Fig 5); furthermore, it intensifies color contrast thereby highlighting the visibility of vessels, while retaining natural color perception (see Fig 2 and Fig 4). These advantanges can be appreciated above all during intraventricular procedures, where neurovascular structures are better depicted (Fig 4). It should be noted that this videoendoscope represents the electronic version of the so-called ENDOCAMELEON (Karl Storz GmbH, Tuttlingen) [12]. The endocameleon works via a fine optomechanical mechanism based on a prism on its tip, while the videoendoscope harbors all the electronic and lighting data in a unique and thin cable, thus resembling a modern "all-in-one" computer technology.

The device offers variable viewing from 0–70° in all directions; its ergonomic shape and the reduced weight render the device ergonomic and well suitable for "so-called" free-hand techniques. This is a marked ergonomic amelioration as compared to the rigid endoscope connected to the HD-camera and light cable. Furthermore, the moving angle of the field of view offers a wide range of freedom, especially during intraventricular procedures. Finally, the ability to set the horizon of vision as desired is a novel and welcomed feature. As a matter of fact, one of the difficulties in performing neuroendoscopy with conventional endoscopes is the positioning of light-post, above all when working with angled scopes. This need is obviated by the integration of the light source into the handpiece, and the ability to "rotate the horizon" via software/camera post-processing. This extra function helps the surgeon to stay oriented while rotating the videoendoscope to reach a specific area of interest with an instrument. Moreover, the use of a light-emitting diode (LED) helps reduce the risk of thermal injuries to surrounding tissue, which can occur when using external Xenon beam light sources.

Visualization has always been a key concept in the development of neurological surgery, serving as either a springboard or limitation for the development of novel techniques. In his text "The Pituitary Body and Its Disorders", Harvey Cushing stressed that "every step of the [neurosurgical] procedure must be conducted under the eye of the operator." The idea of being able to look closely at the surgical target, by means of an optical instrument introduced into the body, was already conceived of by Nitze [13], who built a new tool made of a series of lenses with a source of illumination on the tip and is credited with developing the first modern endoscope.

As a matter of fact, technological innovations such as the introduction of the SELFOC lens, CCDs and fiberoptics significantly contributed to the development of endoscopic neurosurgery. In the last several decades, however, endoscopy has been a major component of advancement in daily neurosurgical practice all over the world, thanks to features such as magnified, panoramic visualization of the relevant anatomy and surgical targets. A variety of neuro-endoscopic routes have now been widely adopted, many of which are replacing or have completely replaced their previous open microsurgical counterparts. Intraventricular neuroendoscopy, skull base endoscopy, spinal endoscopy, and other purely endoscopic and endoscopic-assisted operations are now currently applied.

The endoscope provides a bi-dimensional view and the image as seen on the monitor is the result of a computer elaboration process; loss of spatial and depth information, however, can be overcome with developing experience of the surgeon and also because of the capability of the human brain to elaborate secondary spatial depth cues, e.g., shadows, lights, and parallax movements.

The introduction and evolution of 3D technology in endoscopic surgery has been claimed as a viable solution to overcome limits of 2D vision, although it has not yet become widespread. Current “3D” technology is not strictly 3D, because it provides a complex recombination of two frames of images captured from different angles and requires the use of dedicated glasses to fuse these into a single image. New generation 2D instruments, featuring Ultra-HD (UHD) technology, do not have these requirements.

From a clinical standpoint, neuroendoscopy was vitalized by popularity of the endoscopic third ventriculostomy (ETV) for the treatment of obstructive hydrocephalus, endoscopic marsupialization of arachnoid cysts, and/or colloid cyst resection inside the third ventricle [2]. Neuroendoscopy has also shown great utility in different areas of the brain, outside of the ventricular system. At the present time, it is used for neurosurgical treatment of many diseases, including skull base tumors, vascular lesions, spine and peripheral nerve pathology, and craniosynostosis, because the endoscope has offered the great advantage of reaching deep areas and bringing the surgeon's eyes close to the relevant anatomy, while minimizing brain manipulation and retraction.

During our anatomical lab assessment of the novel system, the rigid, steerable videoendoscope proved to be safe and effective in a standard pure or endoscope-assisted environment. However, there are some limitations and drawbacks that must be considered. First of all, working channels could be added in order to evaluate its efficacy during endoscope-controlled (e.g., endoventricular) procedures. Additionally, because of its integrated design, the system does not seem to be upgradable, thereby mandating replacement of the entire unit if needed. The “all-in-one” technology which makes the system so streamlined may also be a limitation in that the entire unit would be out of service, even in case of failure of a single component. An irrigation sheath to clean the lens of the videoendoscope could be added for endonasal procedures. However, in the present frantic technological revolution era, this may be the price to pay to optimize weight reduction and miniaturization while maintaining image quality and effectiveness.

## Conclusion

The new videoendoscope we tested in a cadaver simulation model, with its steerable variable HD-view and ergonomic design, may one day become an integral component of of the neuroendoscopic armamentarium. The videoendoscope prototype could potentially meet the requirements in the current neuroendoscopic setting and eventually bring with it several advantages to different subspecialties of neurosurgery. Further studies and evaluation of additional clinical applications are needed to properly frame the pros and cons of this new videoendoscope device.
